# Association of Inventory to Measure and Assess imaGe Disturbance – Head and Neck Scores With Clinically Meaningful Body Image-Related Distress Among Head and Neck Cancer Survivors

**DOI:** 10.3389/fpsyg.2021.794038

**Published:** 2021-12-10

**Authors:** David Macias, Brittany N. Hand, Patrik Pipkorn, Amy M. Williams, Steven S. Chang, Joseph Zenga, Marci L. Nilsen, Bethany A. Rhoten, Andrew T. Huang, Nosayaba Osazuwa-Peters, Stacey Maurer, Wendy Balliet, Hong Li, Kenneth J. Ruggiero, Katherine R. Sterba, Evan M. Graboyes

**Affiliations:** ^1^Department of Otolaryngology, Head, and Neck Surgery, Medical University of South Carolina, Charleston, SC, United States; ^2^School of Health and Rehabilitation Sciences, The Ohio State University, Columbus, OH, United States; ^3^Department of Otolaryngology, Head, and Neck Surgery, Washington University School of Medicine, St. Louis, MO, United States; ^4^Department of Otolaryngology, Head, and Neck Surgery, Henry Ford Health System, Detroit, MI, United States; ^5^Department of Otolaryngology and Communication Sciences, Medical College of Wisconsin, Milwaukee, WI, United States; ^6^Department of Otolaryngology, Head, and Neck Surgery, University of Pittsburgh School of Medicine, Pittsburgh, PA, United States; ^7^Vanderbilt University School of Nursing, Nashville, TN, United States; ^8^Bobby R. Alford Department of Otolaryngology, Head, and Neck Surgery, Baylor College of Medicine, Houston, TX, United States; ^9^Department of Head and Neck Surgery and Communication Sciences, Duke University School of Medicine, Durham, NC, United States; ^10^Department of Psychiatry and Behavioral Sciences, Medical University of South Carolina, Charleston, SC, United States; ^11^Department of Public Health Sciences, Medical University of South Carolina, Charleston, SC, United States; ^12^College of Nursing, Medical University of South Carolina, Charleston, SC, United States

**Keywords:** body image distress, disfigurement, head and neck cancer, depression, anxiety, quality of life, patient-reported outcome measure (PROM), survivorship

## Abstract

**Objective:** The Inventory to Measure and Assess imaGe disturbance – Head and Neck (IMAGE-HN) is a validated patient-reported outcome measure of head and neck cancer-related body image-related distress (BID). However, the IMAGE-HN score corresponding to clinically relevant BID is unknown. The study objective is to determine the IMAGE-HN cutoff score that identifies head and neck cancer patients with clinically relevant BID.

**Methods:** We conducted a cross-sectional study at six academic medical centers. Individuals ≥18 years old with a history of head and neck cancer treated with definitive intent were included. The primary outcome measure was the IMAGE-HN. A Receiver Operating Characteristic curve analysis was performed to identify the IMAGE-HN score that maximized sensitivity and specificity relative to a Body Image Scale score of ≥10 (which indicates clinically relevant BID in a general oncology population). To confirm the validity of the IMAGE-HN cutoff score, we compared the severity of depressive [Patient Health Questionnaire-9 (PHQ-9)] and anxiety symptoms [Generalized Anxiety Disorder-7 (GAD-7)], and quality of life [University of Washington-QOL (UW-QOL)] in patients with IMAGE-HN scores above and below the cutoff.

**Results:** Of the 250 patients, 70.4% were male and the mean age was 62.3 years. An IMAGE-HN score of ≥22 was the optimal cutoff score relative to a Body Image Scale score of ≥10 and represents a clinically relevant level of head and neck cancer-related BID. Relative to those with an IMAGE-HN score of <22, patients with IMAGE-HN scores of ≥22 had a clinically meaningful increase in symptoms of depression (mean PHQ-9 score difference = 5.8) and anxiety (mean GAD-7 score difference = 4.1) as well as worse physical (mean UW-QOL score difference = 18.9) and social-emotional QOL (mean UW-QOL score difference = 21.5). Using an IMAGE-HN cutoff score ≥22, 28% of patients had clinically relevant BID.

**Conclusion:** An IMAGE-HN score of ≥22 identifies patients with clinically relevant head and neck cancer-related BID. This score may be used to detect patients who could benefit from strategies to manage their distress, select patients for studies evaluating interventions to manage head and neck cancer-related BID, and improve our understanding of the underlying epidemiology of the disorder.

## Introduction

There are nearly 500,000 head and neck cancer (HNC) survivors in the United States (US) and this population is growing exponentially (Tota et al., 2019; Howlader et al., 2020). Because HNC arises in cosmetically and functionally critical areas, such as the tongue, jaw, throat, and face, patients with HNC experience substantial life-altering morbidity related to disfigurement, difficulty swallowing, impaired smiling, and speaking challenges. As a result, 75% of patients with HNC express body image concerns (Fingeret et al., 2012), and it is estimated that up to 20% meet criteria for body image-related distress (BID) (Melissant et al., 2021a), a disorder characterized by a distressing self-perceived change in appearance and function (Fingeret et al., 2012; Rhoten, 2016; Teo et al., 2016; Ellis et al., 2019b). BID is associated with devastating psychosocial morbidity, such as social isolation, stigmatization, depression, and decreased quality of life (QOL) (Fingeret et al., 2012, 2015; Rhoten et al., 2013). BID, in addition to a number of other factors, contributes to HNC survivors dying from suicide at 2 times the rate of other cancer types and 4 times that of the US general population (Osazuwa-Peters et al., 2018, 2021).

Due to its subjective nature and poor correlation with objective measures of disfigurement (Manier et al., 2018; Graboyes et al., 2020a), BID is best measured using patient-reported outcome measures (PROMs). Unfortunately, the PROMs that have been used to assess BID in patients with HNC have been limited by concerns about construct validity and psychometric performance (Ellis et al., 2019a). The Inventory to Measure and Assess imaGe disturbance – Head and Neck (IMAGE-HN) was created to fill this gap (Graboyes, 2021). IMAGE-HN is a psychometrically valid 24-item PROM developed in accordance with the Patient Reported Outcomes Measurement Information System (PROMIS) guidelines (PROMIS, 2013) to comprehensively assess HNC-related BID (Graboyes et al., 2020a). Although IMAGE-HN underwent rigorous validation in a multi-institutional cohort, the IMAGE-HN score that corresponds to clinically relevant BID remains unknown. As a result, clinicians and researchers are limited in their ability to identify patients with HNC-related BID, preventing appropriate referrals for management of this devastating disorder and enrollment into clinical trials to test the efficacy of novel interventions. Therefore, the objective of this study is to determine the IMAGE-HN score that identifies clinically relevant BID in patients with HNC.

## Materials and Methods

### Study Design and Patients

A cross-sectional study was conducted at six academic medical centers in the US (Medical University of South Carolina, Washington University School of Medicine, the University of Pittsburgh School of Medicine, Vanderbilt University Medical Center, Henry Ford Health System, and the Medical College of Wisconsin). This study was approved by the institutional review board at each institution. Individuals ≥18 years old with a history of HNC (i.e., oral cavity, pharynx, larynx, nose/paranasal sinuses, major salivary gland, or cutaneous structures of the head and neck) who had undergone definitive treatment and were free of known active disease were eligible for the study. Patients were excluded if they were unable to read English. Patients were recruited during routine follow-up or survivorship visits at multidisciplinary head and neck oncology clinics from November 2020 to August 2021 and enrolled face-to-face by a study team member following provision of written informed consent. Following enrollment, patients completed study assessments using an electronic tablet. Of 284 patients approached for participation, 23 declined and 11 did not provide demographic or oncologic data, leaving a sample of 250. Patients were compensated $10 for participation. This study followed the Strengthening the Reporting of Observational Studies in Epidemiology (STROBE) reporting guideline (von Elm et al., 2014).

### Outcome Measures

#### Head and Neck Cancer-Related Body Image-Related Distress

The primary outcome measure was the IMAGE-HN global score. The IMAGE-HN is a 24-item PROM that assesses multiple domains of HNC-related BID including other-oriented appearance concerns, personal dissatisfaction with appearance, distress with functional impairments, and social avoidance and isolation. Global IMAGE-HN scores range from 0 to 84, with higher scores indicating more severe HNC-related BID (Graboyes et al., 2020a). The IMAGE-HN instrument and scoring manual are publicly available (Graboyes, 2021).

#### Legacy Measure of Body Image-Related Distress

The Body Image Scale (BIS) is a 10-item PROM that assesses the affective, cognitive, and emotional aspects of body image due to cancer or its treatment over the prior 7 days (Hopwood et al., 2001). Initially developed for breast cancer patients, the BIS has been widely used to study BID in patients with HNC although it has not been specifically validated in this population (Fingeret et al., 2012; Ellis et al., 2019a). Higher scores indicate greater body image concerns and a BIS score of ≥10 corresponds to clinically relevant BID in general oncology patients (Chopra et al., 2020).

#### Depression

Depression was measured using the Patient Health Questionnaire-9 (PHQ-9), a reliable and validated 9-item measure of depressive symptoms (Kroenke et al., 2001). The PHQ-9 was selected because of its performance in patients with HNC (Shunmugasundaram et al., 2020) and because it is among the measures recommended by the American Cancer Society (ACS) and American Society of Clinical Oncology (ASCO) to screen for depressive symptoms (Andersen et al., 2014; Cohen et al., 2016). Scores range from 0 to 27, with higher scores reflecting more severe depressive symptoms. Established cutoff scores of 5, 10, 15, and 20 indicate mild, moderate, moderately severe, and severe depressive symptoms, respectively. A difference of ≥3–4 points between groups on the PHQ-9 is considered clinically important (Kroenke et al., 2020).

#### Anxiety

The Generalized Anxiety Disorder-7 (GAD-7) is a validated 7-item measure of anxiety symptoms. The GAD-7 was chosen because it is the recommended tool to screen for anxiety symptoms in patients with cancer by the ACS and ASCO (Andersen et al., 2014; Cohen et al., 2016). Scores range from 0 to 21, with higher scores indicating more severe anxiety symptoms. Cutoff scores of 5, 10, and 15 are indicative of mild, moderate, and severe anxiety symptoms, respectively (Spitzer et al., 2006). A difference of ≥3 points between groups on the GAD-7 is considered clinically important (Kroenke et al., 2019).

#### Quality of Life

The fourth version of the University of Washington-QOL (UW-QOL) is an HNC-specific questionnaire with 12 domains (pain, appearance, activity, recreation, swallowing, chewing, speech, shoulder, taste, saliva, mood, and anxiety) that assesses QOL within the past 7 days (Rogers et al., 2002). This tool was chosen as it is one of the most widely used HNC-specific measures of QOL (Pateman et al., 2017). Individual domain questions have between 3 and 6 response options scaled evenly from 0 (worst) to 100 (best), according to the hierarchy of response (Rogers et al., 2002). The global UW-QOL score can be broken into two subscale scores, physical function (domains chewing, swallowing, speech, taste, saliva, and appearance) and social-emotional function (domains anxiety, mood, pain, activity, recreation, and shoulder function) (Rogers et al., 2010). Established cutoff scores are not known. A difference of ≥7 points between groups on the UW-QOL composite score is considered clinically important (Vartanian et al., 2004).

### Other Study Variables

Self-reported sociodemographic characteristics include age, gender, race, ethnicity, marital status, living situation, education, employment, rurality, and insurance coverage. Self-reported oncologic characteristics include tumor subsite, cancer treatment, and type of reconstructive surgery. Time since completion of treatment was collected in months.

### Statistical Analysis

Descriptive statistics (e.g., frequencies and percent for categorical variables, mean, and standard deviation for continuous variables) were used to characterize the sample. To determine the IMAGE-HN cutoff score that represents clinically meaningful HNC-related BID, we performed a Receiver Operating Characteristic (ROC) curve analysis to identify the IMAGE-HN score that maximized sensitivity and specificity relative to a BIS score of ≥10 (which indicates clinically relevant BID in a general oncology population) (Chopra et al., 2020). We did this by selecting the point on the ROC curve that minimized the Euclidean distance to the (0,1) point. To examine the clinical validity of our newly defined IMAGE-HN cutoff score, we compared the severity of associated symptoms of depression and anxiety, and QOL (mean PHQ-9, GAD-7, and UW-QOL scores, respectively) in those with and without HNC-related BID, using independent samples *t*-tests. We used Fisher’s Exact tests to compare these subgroups on the proportions of patients with moderate depressive symptoms (defined as PHQ-9 score of ≥10) and anxiety symptoms (defined as GAD-7 score of ≥10) (Spitzer et al., 2006; Hinz et al., 2016). Statistical analyses were performed using SAS. A two-sided *P* < 0.05 was considered statistically significant.

## Results

### Sample Characteristics

A total of 250 patients were included in the study. Table 1 demonstrates the demographic and clinical characteristics of the cohort as well as the mean IMAGE-HN scores (and SD) for each sociodemographic and treatment-related variable. The mean age (SD) was 62.3 (11.9) years; 94.8% (237/250) were non-Hispanic white, and 70.4% (176/250) were male. The most common HNC subsites were oral cavity (35.2%; 88/250), oropharynx (30.4%; 76/250), and larynx/hypopharynx (14.8%; 37/250). Eighty percent of patients were treated with a surgical-based paradigm (201/250) and 47.8% (96/201) underwent free flap reconstruction. The mean (SD) duration since completion of treatment was 22.5 (26.0) months. Of the study patients, 25.2% (63/250) were from the Medical University of South Carolina; 24.8% (62/250) from the Washington University School of Medicine, and 20.0% (50/250) each from the University of Pittsburgh School of Medicine and Medical College of Wisconsin.

**TABLE 1:** Participant characteristics.

Variable	N (%)	IMAGE-HN Mean ± SD
Years of age, Mean ± SD	62.3 ± 11.9	
**Sex**		
Female	74 (29.6)	20.9 ± 20.9
Male	176 (70.4)	13.4 ± 13.4
**Race**		
White	224 (89.6)	15.0 ± 15.0
African American	20 (8.0)	16.6 ± 16.6
Other	6 (2.4)	34.0 ± 25.8
**Ethnicity**		
Hispanic	2 (0.8)	2.0 ± 2.8
Non-hispanic	237 (94.8)	15.0 ± 16.4
Prefer not to answer	11 (4.4)	31.0 ± 26.6
**Marital status**		
Married/current partner	174 (69.6)	13.6 ± 15.6
Single	34 (13.6)	22.4 ± 21.0
Separated/divorced/widowed	42 (16.8)	18.5 ± 18.6
**Living situation**		
Spouse	167 (66.8)	13.9 ± 15.7
Parents/children/friends/other	45 (18.2)	19.9 ± 22.0
Self	38 (15.0)	17.8 ± 16.0
**Educational attainment**		
Less than high school	11 (4.4)	22.9 ± 24.7
High school graduate	73 (29.2)	17.8 ± 17.9
Some college	68 (27.2)	16.7 ± 17.4
College graduate	56 (22.4)	14.4 ± 17.3
Graduate school	42 (16.8)	9.6 ± 10.9
**Employment**		
Employed (either part or full-time or homemaker)	94 (37.6)	15.5 ± 17.7
Not employed (disability or unemployed)	48 (19.2)	26.3 ± 19.3
Retired	108 (43.2)	10.9 ± 13.4
**Rurality**		
Rural	103 (41.2)	15.8 ± 17.5
Suburban	112 (44.8)	15.0 ± 16.6
Urban	35 (14.0)	17.2 ± 18.2
**Insurance**		
Private	96 (38.4)	14.5 ± 16.6
Medicare	116 (46.4)	14.9 ± 17.3
Medicaid/self-pay/other	38 (15.2)	20.5 ± 17.6
**Tumor location**		
Oral cavity	88 (35.2)	17.6 ± 18.8
Oropharynx	76 (30.4)	10.5 ± 14.4
Larynx/hypopharynx	37 (14.8)	20.5 ± 18.0
Nasal cavity/paranasal sinuses/nasopharynx	13 (5.2)	22.3 ± 11.3
Major salivary gland	12 (4.8)	17.3 ± 13.0
Facial cutaneous malignancy	16 (6.4)	11.7 ± 17.9
Other or unknown	8 (3.2)	14.4 ± 21.9
**Cancer treatment**		
Surgery	68 (27.2)	12.5 ± 16.9
Surgery and adjuvant radiation	77 (30.8)	17.9 ± 18.2
Surgery and adjuvant chemoradiation	56 (22.4)	19.4 ± 18.5
Nonsurgical treatment^a^	49 (19.6)	12.0 ± 12.7
**Reconstructive surgery**		
None	80 (39.8)	10.1 ± 15.3
Other (including local or regional flap)	25 (12.4)	18.3 ± 18.2
Microvascular free flap	96 (47.8)	21.3 ± 18.6
**Osseous microvascular free flap reconstruction**		
No	66 (68.8)	18.7 ± 17.1
Yes	30 (31.2)	26.7 ± 20.6
**Time since completion of treatment**		
0–11 months	111 (43.7)	15.9 ± 16.0
1–5 years	106 (41.7)	15.6 ± 19.0
>5 years	22 (8.7)	12.3 ± 13.9
Unknown	15 (5.9)	19.3 ± 16.8
**Academic medical center**		
Medical University of South Carolina	63 (25.2)	18.7 ± 20.9
Washington University School of Medicine	62 (24.8)	15.0 ± 17.0
University of Pittsburgh School of Medicine	50 (20.0)	13.0 ± 16.1
Vanderbilt Ingram Cancer Center	10 (4.0)	16.2 ± 14.6
Medical College of Wisconsin	50 (20.0)	15.5 ± 14.1
Henry Ford Health System	15 (6.0)	13.3 ± 14.6

*^a^Includes radiation and/or chemotherapy.*

### Clinically Relevant Inventory to Measure and Assess imaGe Disturbance – Head and Neck Score

**FIGURE 1:**
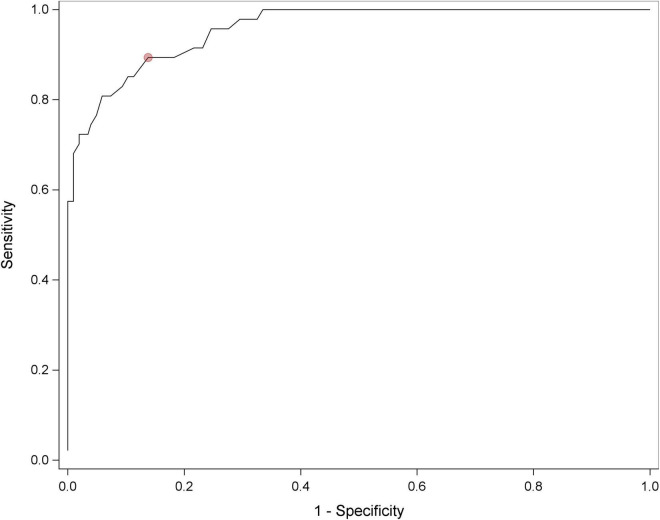
Receiver operating characteristic curve for the IMAGE-HN for identifying clinically relevant HNC-related BID. Area under the curve = 0.96. The red dot represents the IMAGE-HN score that maximized sensitivity and specificity relative to a BIS score of ≥10.

**FIGURE 2:**
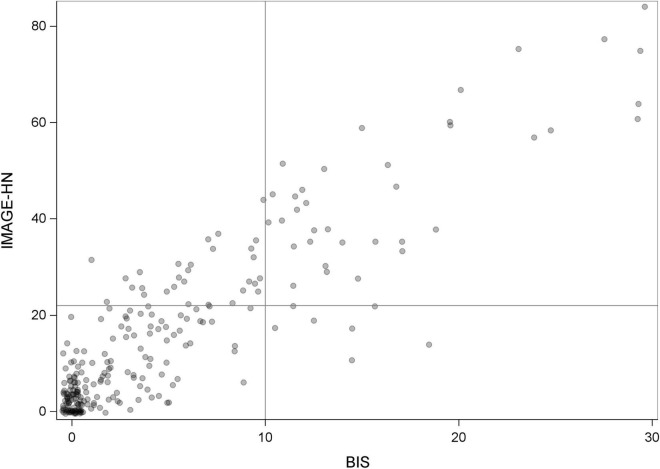
IMAGE-HN vs. BIS scores. Scatterplot showing the distribution of IMAGE-HN and BIS scores for our study sample. Circles represent the IMAGE-HN and BIS scores of each patient, with a jitter (small amount of random noise) applied to minimize overlap of observations. Any remaining overlapping observations are indicated by darker shaded circles. Twenty-eight patients (11% of the cohort) have clinically relevant HNC-related BID as measured by IMAGE-HN score ≥22 who would not be identified using the BIS (BIS < 10; circles in the upper left quadrant). Forty-two patients (17% of the cohort) have clinically relevant HNC-related BID as measured by IMAGE-HN score ≥22 who would have been identified by the BIS (BIS > 10; circles in the right upper quadrant). One hundred seventy-five patients (70% of the cohort) do not have clinically relevant HNC-related BID as measured by IMAGE-HN score < 22, who also have BIS < 10 (bottom left quadrant). Five patients (2% of the cohort) would not be identified as having clinically relevant HNC-related BID as measured by an IMAGE-HN score < 22 who would have been identified using a BIS score > 10 (bottom right quadrant).

An IMAGE-HN score of ≥22 was the optimal dichotomization value relative to a BIS score of ≥10 and represents a clinically relevant level of HNC-related BID (Figure 1). Relative to a BIS ≥10, an IMAGE-HN cutoff score ≥22 was highly sensitive at identifying patients with HNC-related BID (area under curve = 0.96). Overall, 28% of patients with HNC in the cohort (70/250) had clinically relevant BID as determined by an IMAGE-HN score ≥22. An IMAGE-HN score of ≥22 identified 28 additional patients (11% of the study sample) as having clinically relevant BID who would not have been diagnosed with HNC-related BID using the legacy measure (BIS ≥10) (Figure 2; patients in the top left quadrant).

### Association of Clinically Relevant Inventory to Measure and Assess imaGe Disturbance – Head and Neck Scores With Depression, Anxiety, and Head and Neck Quality of Life

Because of the strong association of HNC-related BID with psychological well-being and QOL, we evaluated the association of clinically relevant IMAGE-HN scores with symptoms of depression and anxiety and QOL (Table 2). Relative to those with an IMAGE-HN score of <22, patients with an IMAGE-HN score of ≥22 were more likely to experience more severe depressive symptoms [mean PHQ-9 score = 8.3 (*SD* = 6.2) vs. 2.5 (*SD* = 3.4); mean difference = 5.8] and more severe anxiety symptoms [mean GAD-7 = 6.7 (*SD* = 6.0) vs. 2.6 (*SD* = 3.1); mean difference = 4.1]. These differences between those with and without HNC-related BID are both clinically and statistically significant. Patients were also more likely to experience worse physical QOL [mean UW-QOL physical sub-score = 60.1 (*SD* = 16.8) vs. 79.0 (*SD* = 16.3); mean difference = 18.9] and worse social-emotional QOL [mean UW-QOL social-emotional score = 62.4 (*SD* = 20.6) vs. 83.9 (*SD* = 13.8); mean difference = 21.5; *p* < 0.01 for each]. These differences were also clinically and statistically significant between those with and without HNC-related BID. When analyzed using established cutoff scores for moderate depressive symptoms (PHQ ≥ 10) and moderate anxiety symptoms (GAD ≥ 10), patients with an IMAGE-HN score of ≥22 were more likely to have moderate or severe depressive symptoms (30.0% vs. 5.0%) and moderate or severe anxiety symptoms (24.3% vs. 3.0%; *p* < 0.001 for each) relative to patients with an IMAGE-HN score of <22.

**TABLE 2:** Association of clinically relevant IMAGE-HN scores with depression, anxiety, and head and neck quality of life.

Scale	Full sample (*n* = 250)		IMAGE-HN < 22 (*n* = 180)		IMAGE-HN ≥ 22 (*n* = 70)		*p*-value
	Mean	*SD*	Mean	*SD*	Mean	*SD*	
IMAGE-HN^1^ Global (raw sum)	15.6	17.2	6.8	6.5	38.2	15.3	<0.001
PHQ-9^2^	4.1	5.1	2.5	3.4	8.3	6.2	<0.001
GAD-7^3^	3.4	4.6	2.6	3.1	6.7	6.0	<0.01
UW QOL v4^4^							
Physical	73.7	18.5	79.0	16.3	60.1	16.8	<0.001
Social-Emotional	77.9	18.6	83.9	13.8	62.4	20.6	<0.001

*^1^The Patient Health Questionnaire-9 is a validated measure of depression. Scores range from 0 to 27 with higher scores reflecting more severe depressive symptoms. The minimal important difference is ≥3 to 4 points.*

*^2^The Generalized Anxiety Disorder-7 is a validated measure of anxiety. Scores range from 0 to 21 with higher scores indicating worse anxiety symptoms. The minimal important difference is ≥3 points.*

*^3^The fourth version of the University of Washington-QOL is a HNC-specific questionnaire with 12 domains that assesses quality of life within the last 7 days. Domains are scaled from 0 (worst) to 100 (best) according to the hierarchy of response. The minimal important difference is ≥7 points.*

## Discussion

### Contribution to the Assessment of Head and Neck Cancer-Related Body Image-Related Distress

Although BID has profound consequences in terms of psychosocial well-being and QOL for patients with HNC, HNC-related BID remains poorly understood in large part due to limitations in our ability to measure HNC-related BID and identify patients with this disorder (Rodriguez et al., 2019; Graboyes et al., 2020a). The BIS has been used frequently to assess BID in patients with HNC (Ellis et al., 2019a) and a clinically relevant cutoff score for the BIS was recently determined (Rhondali et al., 2015; Chopra et al., 2020). However, the BIS lacks content validity for HNC-related BID through its (1) omission of key appearance (e.g., drooling and facial asymmetry) and functional (e.g., eating in public and speaking challenges) concerns and (2) inclusion of items not relevant to patients with HNC (e.g., “Did you find it difficult to look at yourself naked?”) (Ellis et al., 2019b). Recently, a number of PROMs have been developed for, and validated among, patients with HNC including the IMAGE-HN, FACE-Q, and McGill Body Image Concerns Scale-Head and Neck Cancer (Cracchiolo et al., 2019; Rodriguez et al., 2019; Graboyes et al., 2020a). The development and validation of each of these HNC-specific PROMs represents significant progress. However, the clinical application of these PROMs to distinguish between HNC patients with and without clinically relevant BID remains uncertain (Macias et al., 2021). The current study addresses this key measurement gap. In a large, multi-institutional cohort, we demonstrated that an IMAGE-HN score of ≥22 represents a clinically relevant threshold and can distinguish between those with and without HNC-related BID.

There are two important caveats to interpreting the optimal IMAGE-HN cutoff score. First, there is no gold standard for the diagnosis of HNC-related BID [e.g., Diagnostic and Statistical Manual of Mental Disorders (DSM) diagnosis] against which to compare the diagnostic accuracy of IMAGE-HN. As a result, the process for determining score thresholds for HNC-related BID is not straightforward. However, the approach to IMAGE-HN development (Ellis et al., 2019b) ensured that we captured relevant conceptual constructs of HNC-related BID (Rhoten, 2016; Melissant et al., 2021a) as well as the associated social, functional, and QOL impairments that are critical to DSM-based diagnoses. Second, our method of determining an IMAGE-HN cutoff score aimed to maximize the sensitivity and specificity of IMAGE-HN relative to the legacy measure (BIS). Another approach would have been to maximize statistical power, or the IMAGE-HN score that maximizes the effect size between those with and without HNC-related BID. Our selected method was optimized to meet the study objective of most accurately identifying patients with clinically relevant BID.

### Epidemiology of Head and Neck Cancer-Related Body Image-Related Distress

Findings from the current study can be applied to better characterize the epidemiology of HNC-related BID. A recent study by Melissant et al. (2021a) estimated that 13–20% of HNC survivors had clinically relevant BID as measured by the BIS. The current study using a HNC-specific measure of BID (IMAGE-HN) shows that (1) 28% of HNC survivors have clinically relevant HNC-related BID and (2) 11% of HNC patients with clinically relevant BID would not have been identified by the BIS. To our knowledge, this is the first study to estimate the prevalence of HNC-related BID utilizing a tool created for and validated in patients with HNC. Future research should utilize the IMAGE-HN and its cutoff score to refine our understanding of the trajectory of HNC-related BID throughout HNC survivorship and better characterize the prevalence of HNC-related BID in relation to demographic, oncologic, and treatment characteristics (Graboyes et al., 2019; Macias et al., 2021).

### Clinical and Research Implications of an Inventory to Measure and Assess imaGe Disturbance – Head and Neck Cutoff Score

A second implication of our study is that the IMAGE-HN cutoff score can now be used in clinical practice and research studies to identify patients with clinically relevant HNC-related BID who might benefit from interventions to manage their distress. A recent national survey showed that body and self-image-related distress was the least likely of all survivorship topics to be addressed by head and neck oncology providers (Cognetti et al., 2020). Prior to this study, clinicians were limited in their ability to identify those with clinically relevant HNC-related BID, inhibiting referrals for further management. Despite the high prevalence of BID in patients with HNC, evidence-based interventions to manage HNC-related BID are lacking (Ellis et al., 2019a; Richardson et al., 2019). Preliminary data from a few recent small studies highlight the promise of a virtually delivered cognitive behavioral intervention (Graboyes et al., 2020b) or a structured expressive writing activity as novel treatments for HNC-related BID (Melissant et al., 2021b). Future research should utilize validated measures of HNC-related BID (e.g., IMAGE-HN) to test interventions aimed at reducing BID in patients with HNC. Furthermore, an IMAGE-HN cutoff score of ≥22 should be utilized as an inclusion criterion for accrual into clinical trials to test the efficacy of interventions intended to decrease HNC-related BID.

### Future Directions

As part of a comprehensive and patient-oriented approach to managing HNC-related BID, the IMAGE-HN can be a powerful tool to help clinicians and patients identify unmet needs. However, there are several areas that still need to be addressed to enhance the clinical relevance of IMAGE-HN. First, the minimal important difference in IMAGE-HN scores over time and between groups are unknown; these values are necessary to evaluate the effectiveness of treatment (Kallogjeri et al., 2020). Second, while a cutoff score for clinically relevant HNC-related BID is an important benchmark, BID likely exists on a continuum and score ranges or thresholds defining disease severity are lacking. Score thresholds can be ascertained using innovative techniques such as bookmarking (Cook et al., 2019) and could guide clinicians and researchers to utilize stepped-therapy approaches that match treatment intensity to severity of BID.

Moreover, factors affecting the clinical implementation of IMAGE-HN are not known. HNC-related BID may be difficult to detect in the clinical setting as symptoms overlap with the adverse effects of cancer-related treatment. In addition, head and neck oncology providers are not readily trained to identify psychosocial concerns, and patients with HNC may be hesitant to express body image-related concerns (Lydiatt et al., 2013). Lessening the shame and embarrassment associated with HNC-related BID is vital to providing high-quality, patient-centered oncology care, which is associated with improved outcomes, including survival, and is prioritized by organizations involved in oncology funding, policy making, and regulation (Basch et al., 2012; Rotenstein et al., 2017). Routine use of IMAGE-HN may help normalize the assessment and treatment of body image concerns in patients with HNC. While the widespread utilization of the IMAGE-HN in busy oncology practices is perhaps unrealistic, adequately screening for and addressing psychosocial concerns in patients with HNC is likely to decrease the overall burden on healthcare resources in the long term by prevention of mental health complications. To improve the clinical implementation of BID screening among patients with HNC, additional research is necessary. This may include studies to identify high-risk groups for targeted screening, leveraging alternative screening tools for distress that assess body image and are already routinely used in the clinical oncology setting (e.g., NCCN Distress Thermometer), or developing a short-form of IMAGE-HN. Finally, even if patients with HNC-related BID are identified in the clinical realm through appropriate screening tools, significant barriers to the delivery and provision of appropriate psychosocial oncology care remains (Pirl et al., 2020). Future research is therefore necessary to investigate the most thoughtful and balanced approach to diagnosing and treating HNC-related BID within our current healthcare delivery models.

### Strengths and Limitations

This study has several strengths. It was conducted with a large sample size from 6 academic medical centers and captured a sample of patients diverse by certain demographic and oncologic characteristics, which enhances the generalizability of study findings. We also used validated PROMs of BID, depression, anxiety, and health related QOL. Finally, we used rigorous statistical methods and incorporated findings from recent advancements in the field of measuring BID among cancer patients to ensure that our cutoff score optimally identifies HNC patients with clinically relevant BID. Despite its strengths, several important limitations should be discussed. Most patients included in this study were white and non-Hispanic, limiting the external validity of the newly defined cutoff score for other races and ethnicities. We only included patients who had completed HNC treatment and were free of active disease. We are thus unable to account for the effect of body image concerns prior to cancer diagnosis and further study of the normative values of IMAGE-HN scores across the trajectory of HNC from diagnosis through treatment should be prioritized. This study relied on self-reported patient characteristics susceptible to recall or response bias. We did not confirm the optimal IMAGE-HN cutoff score in a separate validation cohort. However, this is not expected to be a concern because the study was not attempting to fit a model to our specific sample. Lastly, although no gold standard (e.g., DSM diagnosis) for HNC-related BID exists, the rigorous study methodology we employed ensures the diagnostic accuracy of the IMAGE-HN.

## Conclusion

In this multi-institutional study, an IMAGE-HN score of ≥22 identified patients with clinically relevant HNC-related BID. Furthermore, we found HNC patients with clinically relevant BID suffered clinically meaningful increases in symptoms of depression and anxiety and worse QOL when compared to HNC patients without clinically relevant BID. This score may be used in clinical practice to identify patients with HNC-related BID who may benefit from interventions to manage their distress. Researchers may use the IMAGE-HN cutoff score to improve our understanding of the underlying epidemiology of the disorder and better stratify patients for accrual into clinical trials evaluating novel strategies to manage HNC-related BID.

## Data Availability Statement

The raw data supporting the conclusions of this article will be made available by the authors, without undue reservation.

## Ethics Statement

The studies involving human participants were reviewed and approved by Medical University of South Carolina Institutional Review Board, Washington University School of Medicine Institutional Review Board, University of Pittsburgh School of Medicine Institutional Review Board, Vanderbilt-Ingram Cancer Center Institutional Review Board, Henry Ford Health System Institutional Review Board, and Medical College of Wisconsin Institutional Review Board. The patients/participants provided their written informed consent to participate in this study.

## Author Contributions

DM, BH, and EG: full access to the data in the study and take responsibility for the integrity of the data and accuracy of the data analysis. DM and EG: concept and design and obtained funding. DM, BH, PP, AW, SC, JZ, MN, BR, AH, NO-P, SM, WB, HL, KR, KS, and EG: acquisition, analysis, or interpretation of data and critical revision of the manuscript for important intellectual content. DM and BH: drafting of the manuscript. BH and HL: statistical analysis. All authors contributed to the article and approved the submitted version.

## Conflict of Interest

The authors declare that the research was conducted in the absence of any commercial or financial relationships that could be construed as a potential conflict of interest.

## Publisher’s Note

All claims expressed in this article are solely those of the authors and do not necessarily represent those of their affiliated organizations, or those of the publisher, the editors and the reviewers. Any product that may be evaluated in this article, or claim that may be made by its manufacturer, is not guaranteed or endorsed by the publisher.
